# Room-temperature X-ray fragment screening with serial crystallography

**DOI:** 10.1063/4.0001012

**Published:** 2025-10-27

**Authors:** Sebastian Guenther, Alke Meents

**Affiliations:** DESY, Hamburg, Hamburg, Germany

## Abstract

Most structure determinations using both X-rays and cryo-EM were carried out at cryogenic temperatures. It is therefore very likely that our current picture of protein structure has a bias towards these temperatures and in particular underrepresents energetically higher states. However, physiological processes typically take place at room temperature and above, where energetically higher-lying states and the resulting protein flexibility should play a major role. In addition to the impact on enzyme reactions themselves, this bias should also have a significant influence on ligand binding, as used, for example, in structure-based drug discovery. And indeed, there are some reports of different ligand binding behavior as a function of temperature.

In order to systematically investigate the influence of temperature on ligand-binding in the context of structure-based drug discovery, we therefore carried out the same fragment screen under otherwise identical conditions, once with conventional rotational data collection at 100K and once with the method of fixed-target serial crystallography at room temperature. Serial crystallography should make it possible to significantly reduce the influence of radiation damage and the associated loss of resolution by distributing the dose across thousands of crystals, compared to single- crystal measurements.

We selected the fosfomycin resistance protein FosAKP as target protein and performed a screen against the f2X entry library containing 95 fragments. Interestingly, we were able to achieve almost the same resolution in the serial crystallography experiments at room temperature as with single-crystal measurements at room temperature. Compared to the serial measurements, single-crystal measurements at room temperature provided a 0.3 - 0.4 angstrom worse resolution.

The presentation will cover both the methodological aspects of fixed-target serial crystallography at room temperature and also discuss the results of the screen at the two different temperatures in relation to structure-based drug discovery.

